# Knowledge evolution in physics research: An analysis of bibliographic coupling networks

**DOI:** 10.1371/journal.pone.0184821

**Published:** 2017-09-18

**Authors:** Wenyuan Liu, Andrea Nanetti, Siew Ann Cheong

**Affiliations:** 1 School of Physical and Mathematical Sciences, Nanyang Technological University, 21 Nanyang Link, Singapore 637371, Republic of Singapore; 2 School of Art, Design and Media, Nanyang Technological University, 81 Nanyang Drive, Singapore 637458, Republic of Singapore; 3 Complexity Institute, Nanyang Technological University, 18 Nanyang Drive, Singapore 637723, Republic of Singapore; Dalian University of Technology, CHINA

## Abstract

Even as we advance the frontiers of physics knowledge, our understanding of how this knowledge evolves remains at the descriptive levels of Popper and Kuhn. Using the American Physical Society (APS) publications data sets, we ask in this paper how new knowledge is built upon old knowledge. We do so by constructing year-to-year bibliographic coupling networks, and identify in them validated communities that represent different research fields. We then visualize their evolutionary relationships in the form of alluvial diagrams, and show how they remain intact through APS journal splits. Quantitatively, we see that most fields undergo weak Popperian mixing, and it is rare for a field to remain isolated/undergo strong mixing. The sizes of fields obey a simple linear growth with recombination. We can also reliably predict the merging between two fields, but not for the considerably more complex splitting. Finally, we report a case study of two fields that underwent repeated merging and splitting around 1995, and how these Kuhnian events are correlated with breakthroughs on Bose-Einstein condensation (BEC), quantum teleportation, and slow light. This impact showed up quantitatively in the citations of the BEC field as a larger proportion of references from during and shortly after these events.

## Introduction

According to Karl Popper, science progresses through repeated hypothesis testing [1]. Hypotheses contrary to empirical evidence must be rejected, while those consistent with data survive to be tested another day. In this picture of the scientific enterprise, our knowledge of the world around us is always tentative, but becomes more complete over time. On the other hand, Thomas Kuhn believes that the accepted knowledge of a given time is the result of consensus amongst scientists, based on evidences consistent with their theories [2]. However, when too many conflicting evidences are found, a new consensus can form around new theories in what he called a “paradigm shift”. Kuhn gives special relativity and quantum theory as examples of paradigm shifts. Looking back, we realize these two theories have enormous impacts on how we understand the world today. But could there be paradigm shifts of various scales that have also contributed to reshaping our knowledge of physics?

Many historians of science have noted the strongly reductionistic flavor of scientific research in the last couple of centuries [3]. Starting as natural philosophy, the body of scientific knowledge became separated disciplines of astronomy, biology, chemistry and physics. Within physics itself, we also observe the emergence of high energy physics, condensed matter physics, biophysics, and photonics. These are the results of the splitting of science into more specialized fields. We also observe in parallel the merging of fields, such as the merging of astronomy and physics to give astrophysics, biology and chemistry to give biochemistry, and others “that arose by division and recombination of specialties already matured” [2]. These developments have been discussed extensively by philosophers and historians of science, but unlike our quantitative understanding of physics, our appreciation for the processes through which we acquired our knowledge of physics remains at a highly descriptive level. Some progress has been made in addressing this problem [4–6]. In particular, the following three papers provide the inspiration for our study. Chen and Redner suggested that long-range connections can form between disparate fields because of the development of “a widely applicable theoretical technique, or cross fertilization between theory and experiment” [7]. Visualizing the cross citations between neuroscience journals, Rosvall and Bergstrom traced the growth and maturation of neuroscience as a discipline [8]. Using embryology as a specific example, Chavalarias and Cointet created a phylomemetic network visualization for the evolution of science [9].

## Materials and methods

**Fig 1:**
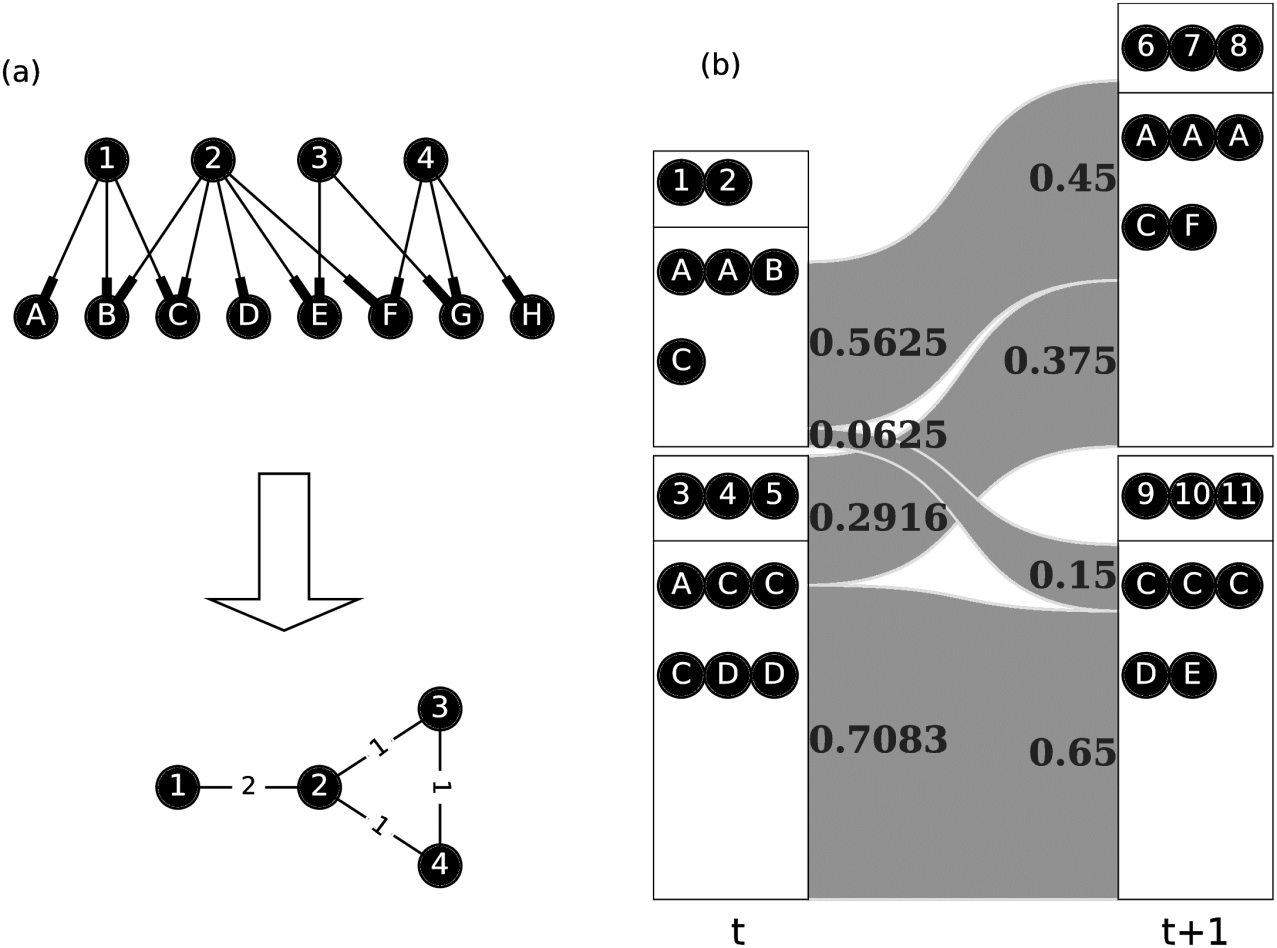
(a) Building a BCN (lower) from a citation network (upper): circles with numbers are papers under consideration, circles with letters are their references, and numbers on edges are weights. (b) Topical clusters (papers 1, 2 citing reference A twice, reference B once, and reference C once; and papers 3, 4, 5 citing reference A once, reference C three times, and reference D twice) in year *t* (left) and (papers 6, 7, 8 citing reference A three times, reference C once, and reference F once; and papers 9, 10, 11 citing reference C three times, reference D once, and reference E once) in year *t* + 1 (right), and their forward (right of the year-*t* TCs) and backward (left of the year-*t* + 1 TCs) intimacy indices, shown as flows.

While these previous studies point to the evolution of scientific knowledge, they do not identify the entity that is recognizably ‘knowledge’, or they do not study the interactions between such objects. To clarify what constitutes knowledge, we start with the bibliographic coupling network (BCN) [10], proposed by Kessler and used extensively in computer science [11, 12]. In a BCN, nodes represent papers, and if two papers share *w* common references, we draw an edge with weight *w* between them (see Fig 1(a)). The BCN is suitable for our purpose for two reasons: (i) the BCN for a given year consists only of papers published that year and does not change after more papers are published later, so features in the BCN represent the state of knowledge in that year; and (ii) the appropriate collective unit of knowledge is a community in the BCN instead of a few key papers or a journal. The data we used in this study is the APS data set, consisting of about half a million publications between 1893 and 2013 [13].

**Fig 2:**
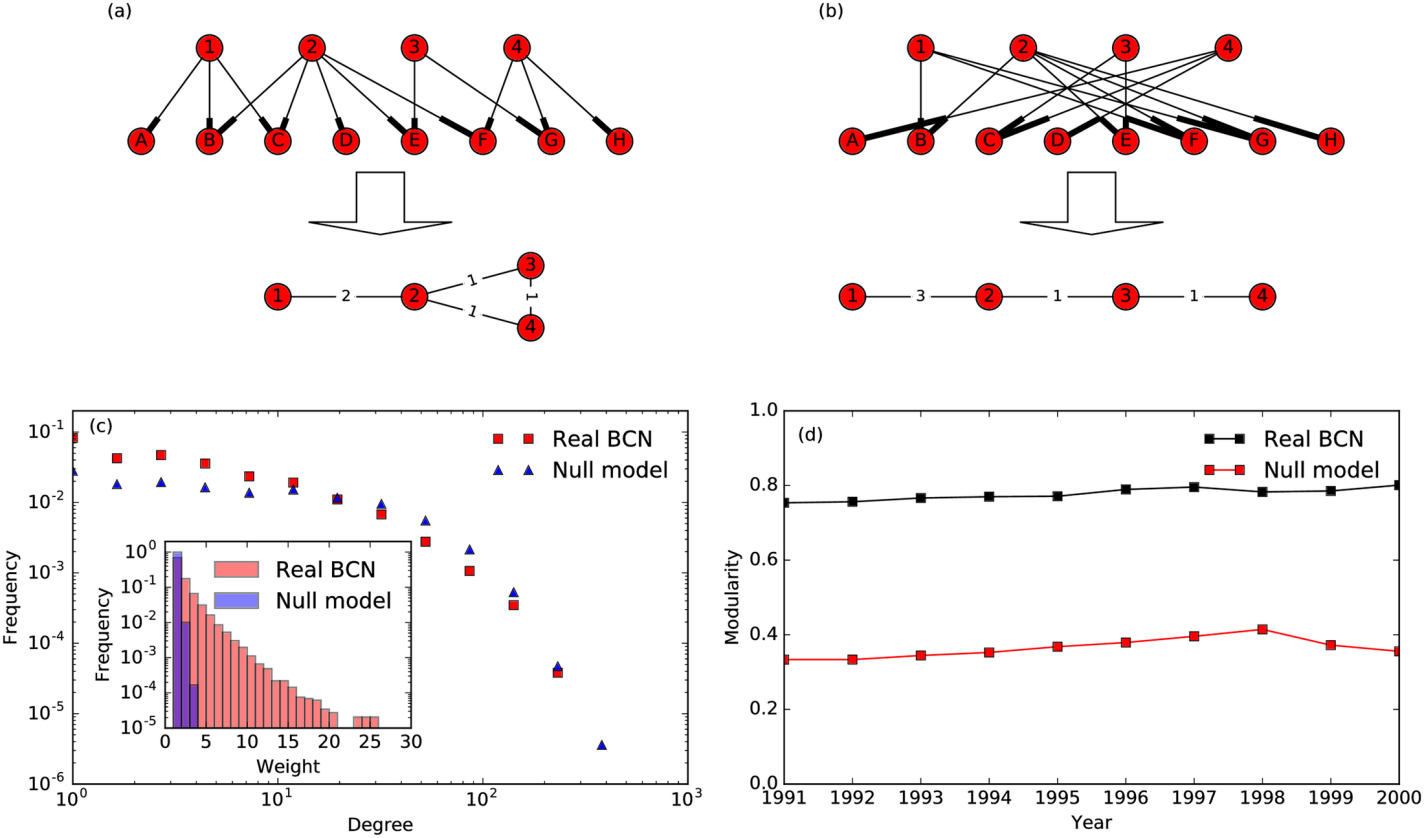
(a) Original citation network and its BCN. (b) A rewired citation network keeping in degrees and out degrees fixed and its BCN. (c) Comparison of the degree and weight distributions of papers published in 1991, between the real BCN and the null model. (d) Modularities of the best partitions extracted by the Louvain method for the real BCNs and the null model between 1991 and 2000. Results from null model are averaged over 10 different rewirings, and the error bars are much smaller than the marker size.

To determine the statistical significance of our empirical BCNs (Fig 2(a)), we build a null model for comparison. In our null model, we fix the out degrees and in degrees of all papers (citing and cited), so that we can directly compare the null model to the empirical BCN. We then randomly rewire the edges of the empirical BCN, to get an ensemble of artificial BCNs (Fig 2(b)). If the empirical BCN is obtained purely by chance, its distribution of edge weights should be close to the distribution of edge weights of the ensemble of artificial BCNs. The results show that in the real BCN edge weights are far more heterogeneous than expected from an appropriate null model, suggesting that these weights are meaningful, and not the result of purely random connections.

After building the BCN, we applied the Louvain method based on the maximization of modularity, to extract the community structure [14]. In the Louvain method, we first identify small groups by optimizing the modularity locally on all nodes. Each small group is then treated as a single node, and the local modularity optimization repeated until the modularity cannot be increased by any further clustering. To verify that the communities extracted are really focussed on closely related questions, we check the Physics and Astronomy Classification Scheme (PACS) numbers of members of the communities. Such numbers are provided by the authors to indicate the subfields of physics to which their papers belong. In our case, we only use the first two digits of the PACS numbers, as a balance between accuracy and coverage. To test whether the PACS numbers appearing in the communities could have occurred by chance, we choose one year *t*, build its BCN, extracting the community structure with sizes {*s*_1_, *s*_2_, …, *s*_*n*_}, and then randomly assign papers in year *t* into *n* pseudo-communities of the same sizes, to remove any potential size effects. For a community of size *s*, we then identify the largest subset of papers sharing the same PACS number. This PACS number can represent the subfield of the community to a certain extent, and the fraction of papers in the largest subset reflect the homogeneity of the community. The results show that the communities extracted are papers really focused on closely related topics, so we refer to these validated units of knowledge as *topical clusters* (TCs).

To study how knowledge evolves, we investigate how TCs $\left\{C^{t}\right\}$ in year *t* become $\left\{C^{t+1}\right\}$ in year *t* + 1. The papers published in different years are distinct, but they do overlap in their references. Therefore we use this fact to define a *forward intimacy index* $I_{mn}^{f}$ and a *backward intimacy index* $I_{mn}^{b}$:

$$\begin{array}{c} \begin{array}{cc} I_{mn}^{f} & =\sum_{i}\frac{N(R_{i},R_{n}^{t+1})}{N(R_{i},R^{t+1})}\frac{N(R_{i},R_{m}^{t})}{L(R_{m}^{t})};\\ I_{mn}^{b} & =\sum_{i}\frac{N(R_{i},R_{m}^{t})}{N(R_{i},R^{t})}\frac{N(R_{i},R_{n}^{t+1})}{L(R_{n}^{t+1})}, \end{array} \end{array}$$ (1)

to quantify how close $C_{m}^{t}$ is to $C_{n}^{t+1}$. Here the TCs at *t* and *t* + 1 are $C^{t}=\{C_{1}^{t},...,C_{m}^{t},...,C_{u}^{t}\}$ and $C^{t+1}=\{C_{1}^{t+1},...,C_{n}^{t+1},...,C_{v}^{t+1}\}$, and we denote the references cited by papers in $C_{m}^{t}$ and $C_{n}^{t+1}$ as $R_{m}^{t}=R\left(C_{m}^{t}\right)=[R_{m1},...,R_{mp}]$ and $R_{n}^{t+1}=R\left(C_{n}^{t+1}\right)=[R_{n1},...,R_{nq}]$; and $R^{t}=\{R_{1}^{t},...,R_{m}^{t},...\}$. *N*(*element*, *list*) is the number of times *element* occurs in *list*, and *L*(*list*) is the length of *list*. Both forward and backward indices take on values between 0 and 1. The larger the intimacy index, the clearer the inheritance relationship between two TCs. In this definition, we assume each citation instance in *t* will be uniformly distributed over all instances of the same citation in *t* + 1, while each citation in *t* + 1 receives equal contributions from all instances of the same citation in *t*. In general, this index is asymmetric, i.e. $I_{mn}^{f}\neq I_{mn}^{b}$, because the references are not cited the same number of times in the two years. Take Fig 1(b) for example, to calculate the intimacy indices between ①② and ⑥⑦⑧, we observe that: N(A,R(⑥,⑦,⑧))=3, N(A,R(①,②))=2, N(A,R(⑥,⑦,⑧,⑨,⑩,⑪))=3, L(R(①,②))=4, N(C,R(⑥,⑦,⑧))=1, N(C,R(①,②))=1, N(C,R(⑥,⑦,⑧,⑨,⑩,⑪))=4. Therefore, the forward intimacy index between ①② and ⑥⑦⑧ is $I^{f}=\frac{N(A,R(⑥,⑦,⑧))}{N(A,R(⑥,⑦,⑧,⑨,⑩,⑪))}\times \frac{N(A,R(①,②))}{L(R(①,②))}+\frac{N(C,R(⑥,⑦,⑧))}{N(C,R(⑥,⑦,⑧,⑨,⑩,⑪))}\times \frac{N(C,R(①,②))}{L(R(①,②))}=\frac{3}{3}\times \frac{2}{4}+\frac{1}{4}\times \frac{1}{4}=0.5625$. In a similar way, the backward intimacy index between ①② and ⑥⑦⑧ is $\frac{2}{3}\times \frac{3}{5}+\frac{1}{4}\times \frac{1}{5}=0.45$.

## Results and discussion

According to the above mentioned methodology we visualize the sequence of TCs and their intimacy indices, the evolution of physics research they represent in the form of alluvial diagrams. Before that, we first discuss the TC validation results. As shown in Fig 2(c), in the BCN edge weights are far more heterogeneous than expected from an appropriate null model, This heterogeneity can be explained by the presence of communities that we extracted using the Louvain method. Compared to the null model, the real BCN has more high-weight edges. We suspect these are the most meaningful edges, arising from the paper’s content. If two papers focus on close topics, they will likely have high chance to have more than one common reference, and this effect also manifest itself in the degree distribution: the null model has a flatter degree distribution at small degrees because the edges are drawn by chance, whereas in the real BCN this coupling is based on content, meaning that papers will have edges mostly with papers that are trying to solve the same problems, so the real BCN will have more low-degree nodes, fewer high-degree nodes compared the null model. The most prominent feature of this content-sensitive citation is community structure: in the real BCN, papers focussed on the same topic share more common references with each other than papers focussed on different topics, so that the densities of edges within topics are much higher than between topics. Therefore the modularities of communities extracted by the Louvain method in the real network is much higher than in the null model, as shown in Fig 2(d).

**Fig 3:**
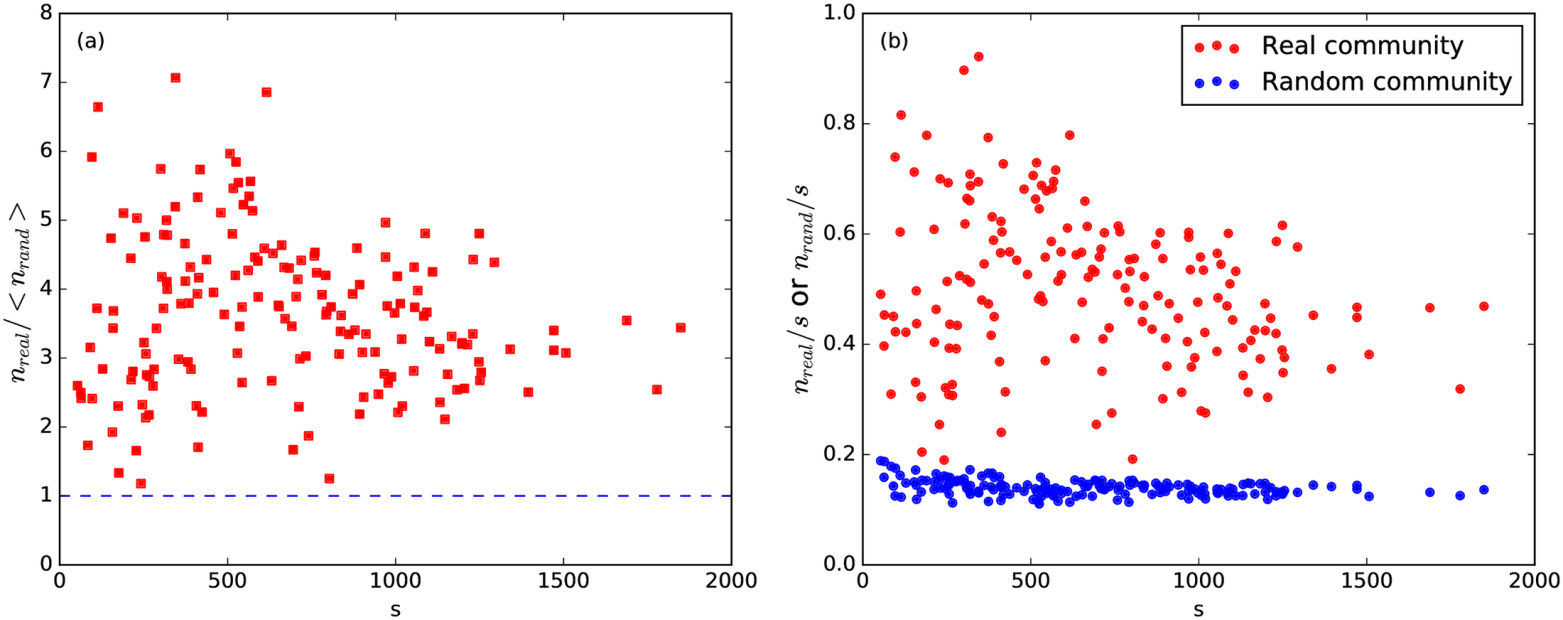
Comparison of PACS homogeneity between real BCN communities between 1991 and 2000 with more than 50 papers, and their corresponding random collections. (a) The red squares correspond to the sizes of the largest subsets of papers sharing at least one PACS number, *n*_*real*_, in the empirical communities divided by the same quantity found in the corresponding random collections, *n*_*rand*_, as a function of the community size *s*. (b) The fraction of the largest subset of papers sharing at least one PACS number as a function of *s* for real communities in the BCN and random collections. For clarity, the small error bars are not shown in the figures.

Meanwhile, We also test how likely the most common PACS number in a community of *n* papers can appear with its observed frequency, within random collections of *n* papers. The largest subset of papers sharing the same PACS number in a random collection of *s* papers is typically small. Dividing the sizes of the largest subsets in the empirical communities and in the random collections, we find ratios are larger than 1 for most cases. That is to say, for most communities, this is highly unlikely, so we conclude that the groupings of papers extracted are meaningful. (see Fig 3).

**Fig 4:**
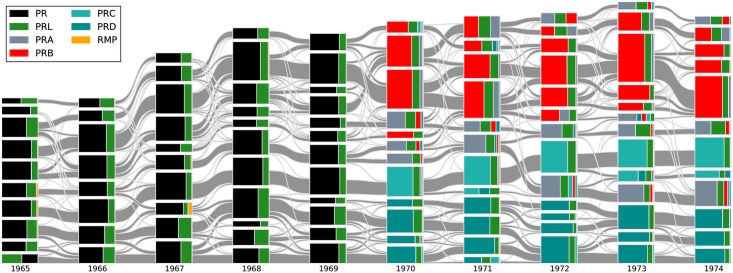
The alluvial diagram of APS papers from 1965 to 1974. Each block in a column represents a TC and the height of the block is proportional to the number of papers in the TC. Only communities comprising more than 100 papers are shown. TCs in successive years are connected by streams whose widths at the left and right ends are proportional to the forward and backward intimacy indices. The different colors in a TC represent the relative contributions from different journals.

The alluvial diagrams can visualize the sequence of TCs and their intimacy indices, the evolution of physics research they represent very clearly. For example, in Fig 4 we can clearly see the birth of PRA, PRB, PRC and PRD from PR in 1970. Each journal consist of several TCs, which existed even in the PR era. The editorial decision to split PR is consistent with the self-organized TCs even though it was done without classification analysis.

**Fig 5:**
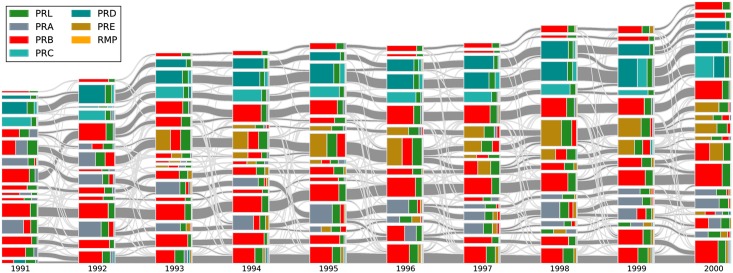
The alluvial diagram of APS papers from 1991 to 2000. Each block in a column represents a TC and the height of the block is proportional to the number of papers in the TC. Only communities comprising more than 100 papers are shown. TCs in successive years are connected by streams whose widths at the left and right ends are proportional to the forward and backward intimacy indices. The different colors in a TC represent the relative contributions from different journals.

We also plotted an alluvial diagram for 1991 to 2000, showing the splitting of PRA into PRA and PRE. As we can see from Fig 5, before 1993, there were several PRA-dominated TCs. After the split in 1993, some PRA-dominated TCs remained PRA-dominated, whereas other PRA-dominated TCs became PRE-dominated. This means that even before 1993, papers in PRA were already divided into groups based on different topics, some of which are predecessors of the PRE TCs.

**Fig 6:**
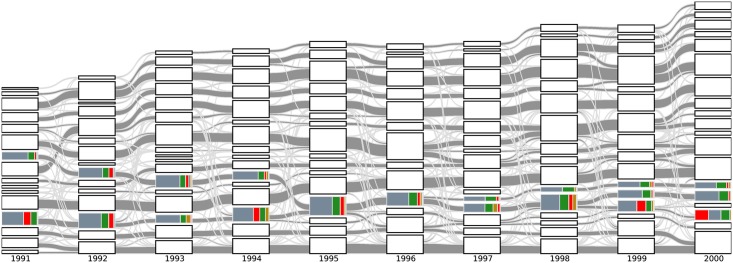
The alluvial diagram of APS papers from 1991 to 2000, where we colored only TCs highly related to quantum optics, quantum information and Bose-Einstein condensation.

More importantly, from the alluvial diagram we can identity the key interactions between TCs that are correlated with important publications. Here we showcase one such episode between 1991 and 2000, involving interesting interactions between quantum optics (QO), quantum information (QI), and Bose-Einstein condensation (BEC). These three fields experienced breakthroughs in the 1990s. In Fig 6 we highlight the evolution of TCs that are related to these three topics and show the three most cited papers in these TCs in Supporting Information S1 File. At the beginning of the decade, we see two PRA-dominated TCs. Based on the papers they contain, we can loosely associate one with quantum information (QI) and trapped atomic ions (BEC), and the other with quantum optics (QO). In 1993, the QI + BEC TC cited many QO papers, and in 1994, the QO TC cited many QI + BEC papers. Following this ‘cross-fertilization’, the two TCs merged in 1995, the same year Cornell *et al*. [15] and Ketterle *et al*. [16] published their seminal papers demonstrating BEC in dilute atomic gases. In recognition of their works, Cornell, Wieman, and Ketterle were awarded the 2001 Nobel Prize in Physics. The PRA-dominated TC split after 1996 to give one that is exclusively BEC, and another that is still a combination of QI + QO. It was after Zeilinger demonstrated in 1997 experimental quantum teleportation [17] that the QI + QO TC split into a QI TC and a QO TC. After receiving more influence from other PRB-dominated TC, the QO cluster produced yet another breakthrough paper, in the form of ultraslow light in hot atomic gases [18]. Without the data visualization done here, few may suspect the existence of such connections between BEC, quantum teleportation and slow light.

**Fig 7:**
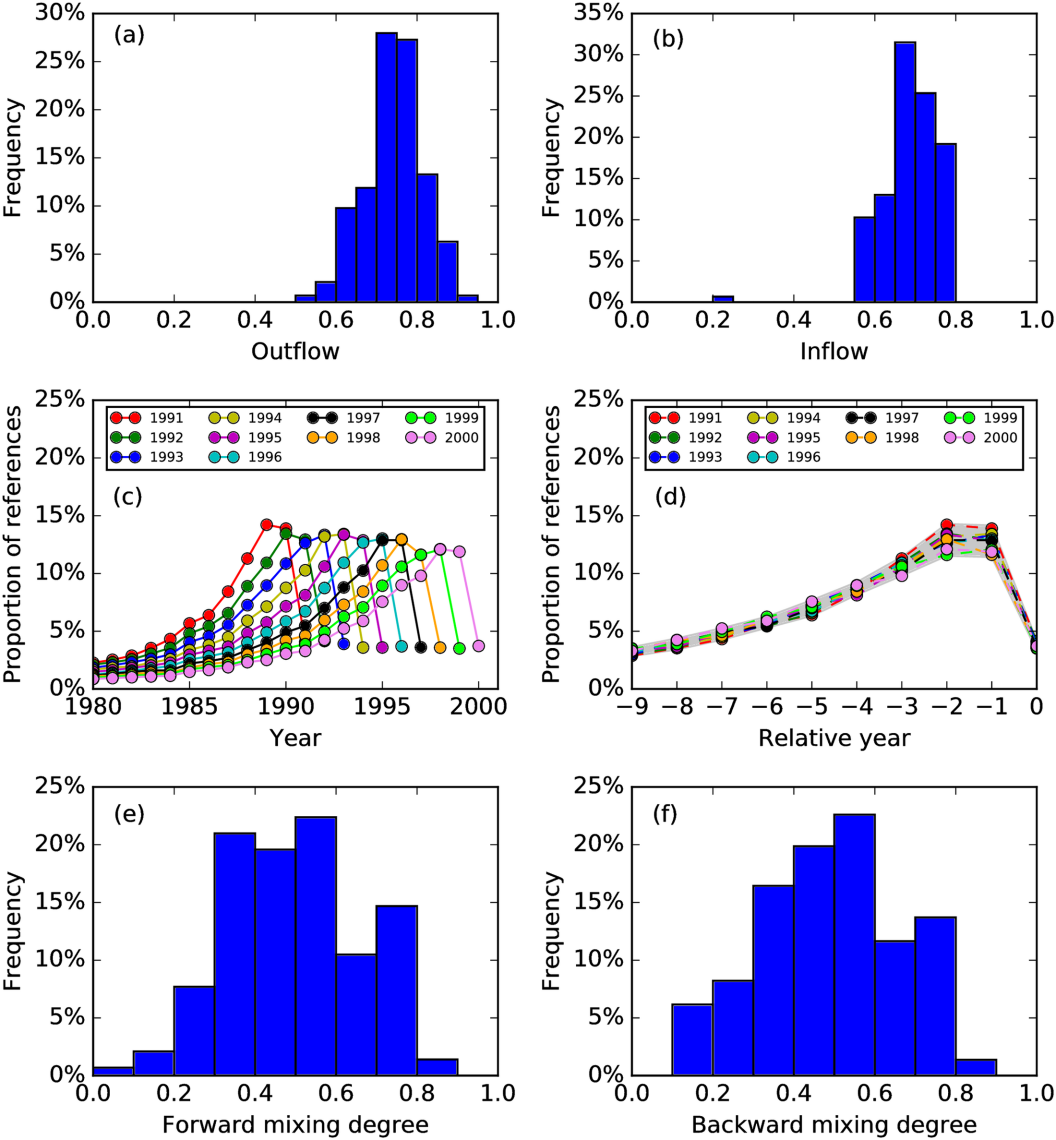
The metabolic analysis of APS papers in the 1990s. (a) The distribution of outflows of TCs. (b) The distribution of inflows of TCs. (c) Proportions of APS paper’s references published in different years. (d) Proportions of APS paper’s references published in different years, relative of the year (0) of publication. (e) The distribution of forward mixing degree of TCs. (f) The distribution of backward mixing degree of TCs.

Some TCs have more references overlapping with those in the previous year, while other TCs have less. To quantify the evolution of references, we sum the forward and backward intimacy indices for each TC. These represent the percentage of a TC’s references going to the next year, and the percentage of references the TC inherited from the previous year, which we think of as the ‘outflow’ and ‘inflow’ respectively. As shown in Fig 7(a) and 7(b), most outflows and inflows are distributed within a narrow range, but there are exceptional cases as well: such as a single peak in Fig 7(b), whose references overlap significantly less than normal with the previous year. In the context of birth, death, growth, decay, split, and merge knowledge processes, we are inclined to call this event in 1993 the birth of a TC. Further analysis shows that most common PACS codes are: 03 (Quantum mechanics, field theories, and special relativity), 42 (Optics) and 63 (Lattice dynamics). Looking at the references of this TC, we find that most of these comes from 1990, 3 year before. This interesting phenomenon is therefore more appropriately identified as a sleeping beauty [19].

**Fig 8:**
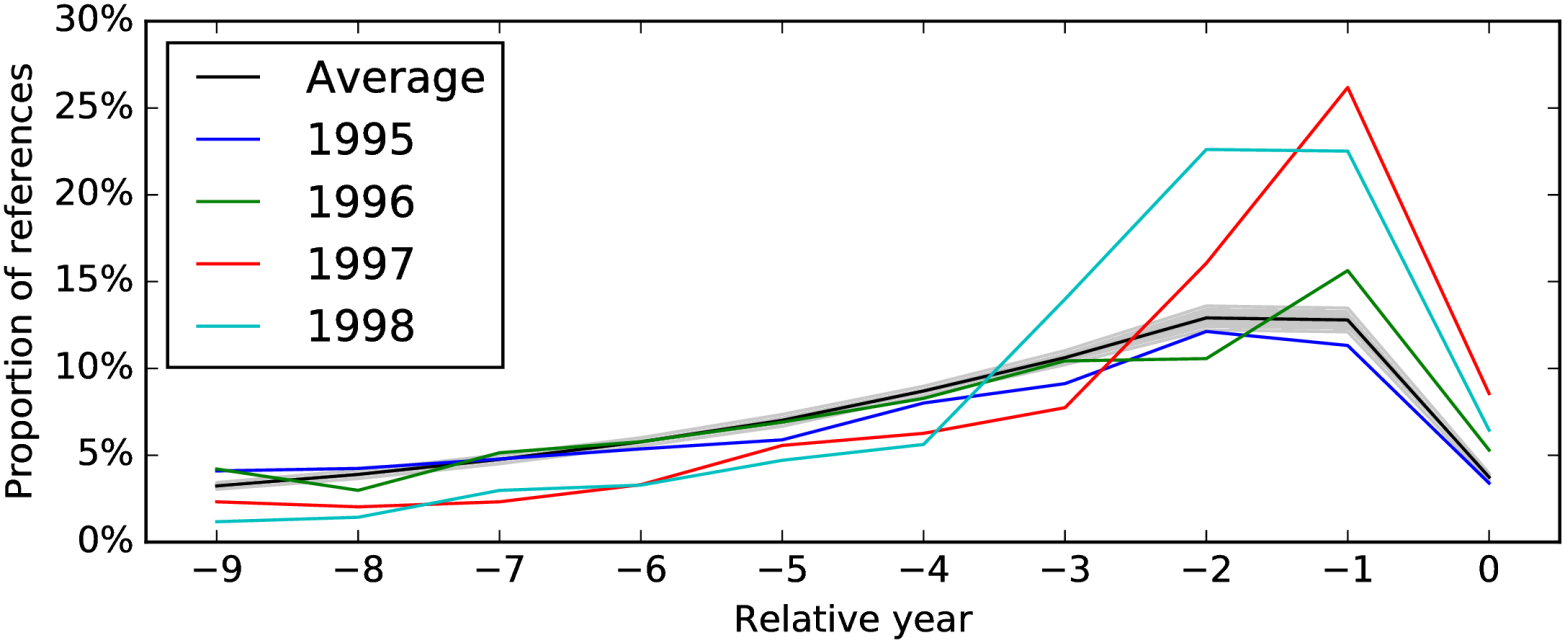
Proportions of a TC’s references published in different years, relative to the year (0) of the TC. The black solid line is the proportions averaged over all TCs in the 1990s, while the area shaded gray is up to one standard deviation away from the mean. Other color lines represent the distribution of four different BEC related TCs.

Every year, physicists absorb new references and drop old references as their fields progress. Although this ‘metabolism’ differ from TC to TC, the whole process is quite stable over all TCs, as shown in Fig 7(c) and 7(d). This universal curve can be used as a benchmark for the test of scientific impact, as we have done in Fig 8.

From Fig 4 we see a diversity of inflows and outflows from one TC to another: some TCs are derived almost exclusively from one source, others receive strong contributions from a small number of sources, or weak contributions from a large number of sources. To quantify such diversity, we construct a forward mixing degree of community $C_{m}^{t}$ and backward mixing degree of $C_{n}^{t+1}$ analogous to the Gini-Simpson index [20]:

$$\begin{array}{cc} M_{m}^{f} & =1-\sum_{n}{\left(I_{mn}^{f}/\sum_{n^{\prime }}I_{mn^{\prime }}^{f}\right)}^{2},\\ M_{n}^{b} & =1-\sum_{m}{\left(I_{mn}^{b}/\sum_{m^{\prime }}I_{m^{\prime }n}^{b}\right)}^{2}, \end{array}$$ (2)

which measure the probabilities that two streams taken at random from the TC’s outflow/inflow (with replacement) represent different streams. A TC with low forward/backward mixing degree has effectively one child/parent, whereas a TC with high forward/backward mixing degree undergoes/results from strong splitting/merging. As shown in Fig 7(e) and 7(f), neither are frequent. It is more common to find weak mixing between TCs, which we believe is due to most papers citing small numbers of papers outside their fields.

At this point, let us recall the Popperian and Kuhnian pictures of the evolution of knowledge, where we expect incremental growth punctuated by abrupt paradigm shifts. Certainly, at the aggregate level of PR series of premier physics journals, the number of articles published has grown over the years. When we partition these articles into TCs, we naively expect that some clusters will grow/shrink because of growing/declining interest in their topics. From the alluvial diagrams, we realize that the real picture is far more complex because of recombinations between TCs. Therefore, instead of measuring the growth rates of pure TCs, we need to measure the growth of recombined TCs. To do this, we assume that the contribution of $C_{m}^{t}$ to the size of $C_{n}^{t+1}$ is proportional to the size of $C_{m}^{t}$ and also the normalized forward intimacy index $I_{mn}^{f}/\sum_{n}I_{mn}^{f}$, i.e.

$$\begin{array}{c} L^{\prime }\left(C_{n}^{t+1}\right)=\sum_{m}L\left(C_{m}^{t}\right)\left(I_{mn}^{f}/\sum_{n}I_{mn}^{f}\right). \end{array}$$ (3)

**Fig 9:**
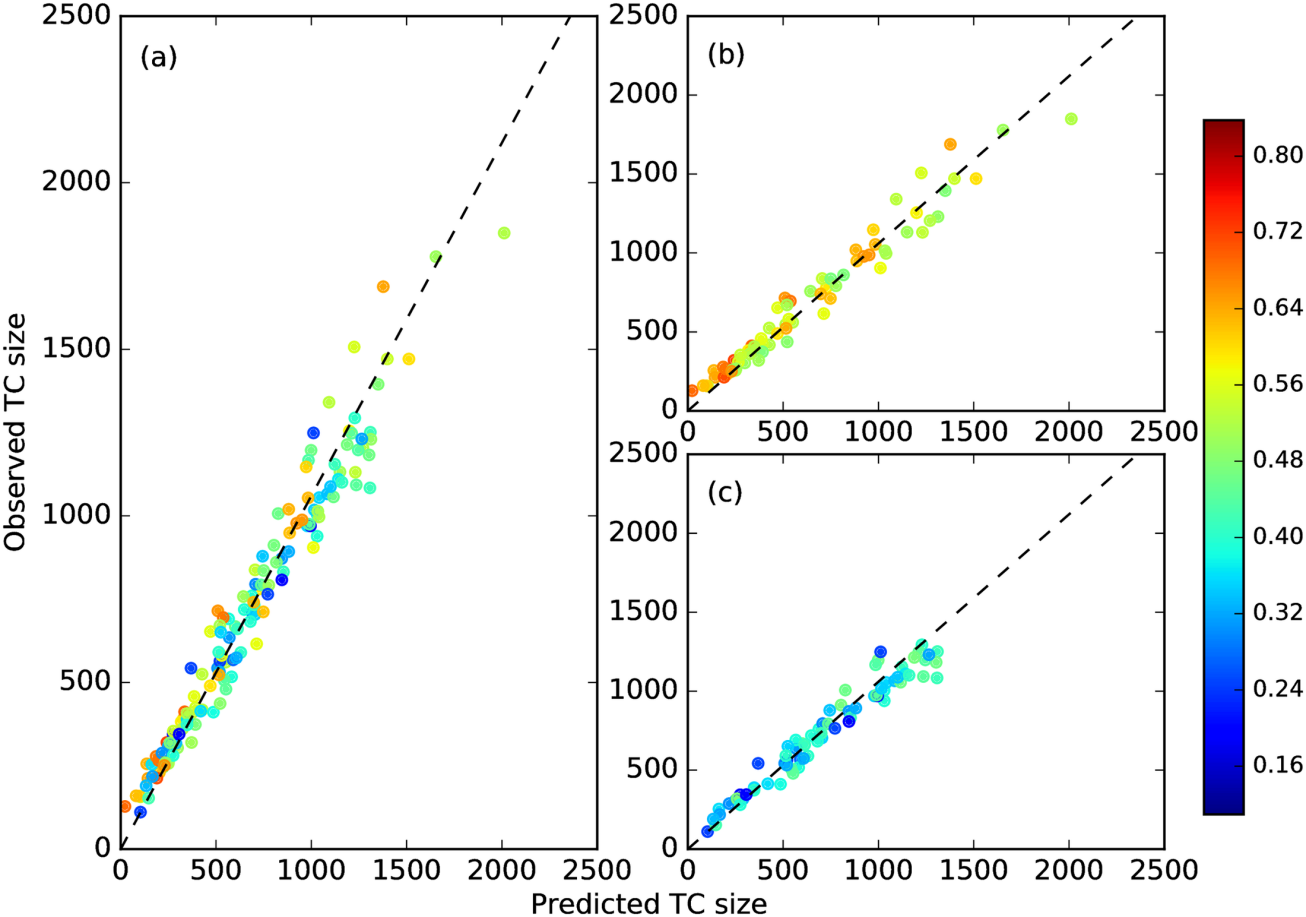
(a) Plot of observed (y-axis) against predicted (x-axis) sizes of recombined TCs, showing a linear growth with slope 1.06 (dashed line). This linear growth is the same for TCs with (b) high (red) or (c) low (blue) backward mixing degree.

When we plot the predicted sizes $L^{\prime }\left(C_{n}^{t+1}\right)$ against the observed size $L(C_{n}^{t+1})$ in Fig 9, we find $\left(L^{\prime }\left(C_{n}^{t+1}\right),L\left(C_{n}^{t+1}\right)\right)$ scattered about a straight line with slope with 1.06, which is the annual growth rate of the number of papers in APS journals. This tells us that the growth of recombined TCs is also Popperian.

**Fig 10:**
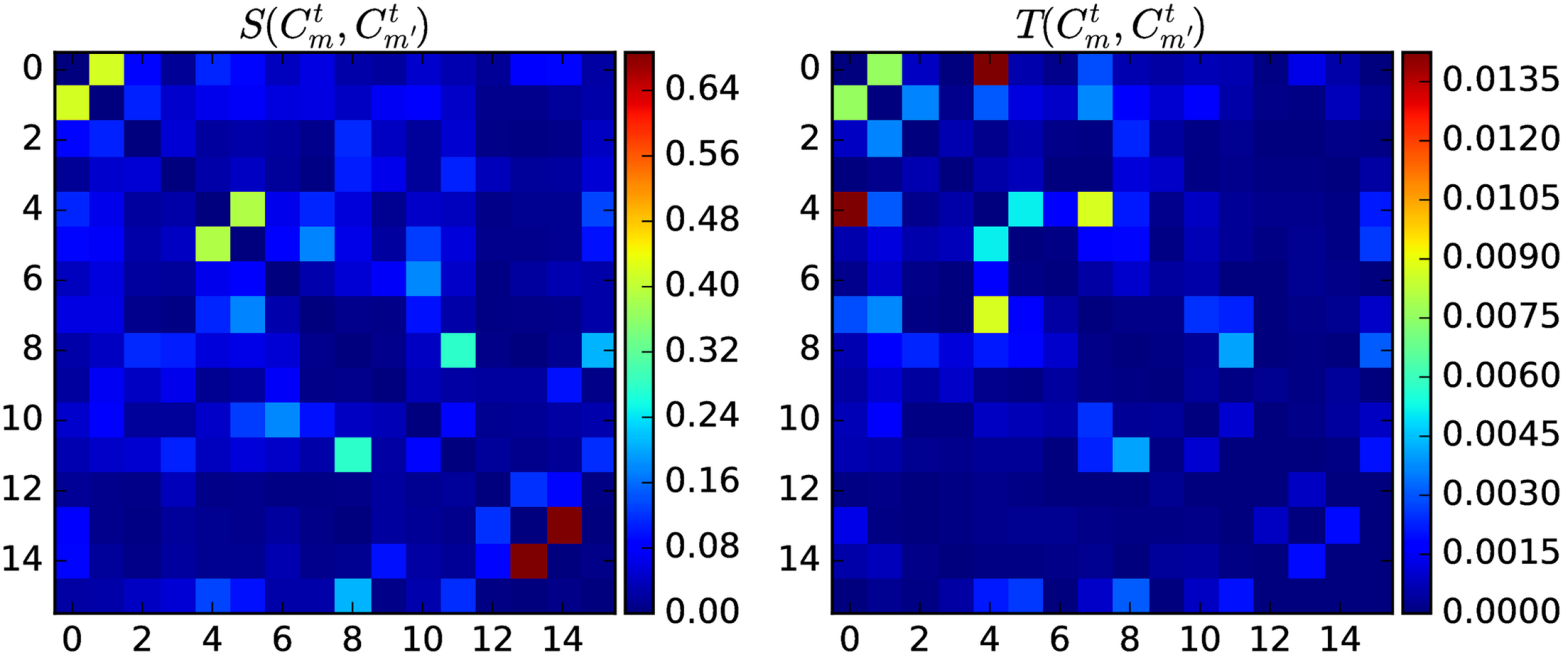
(left) $S(C_{m}^{t},C_{m^{\prime }}^{t})$ of 16 TCs in 1991, computed using forward intimacy indices going from 1991 to 1992. (right) $T(C_{m}^{t},C_{m^{\prime }}^{t})$ of the same 16 TCs, using information from 1991 only. We use the same ordering of TCs in both matrices.

**Fig 11:**
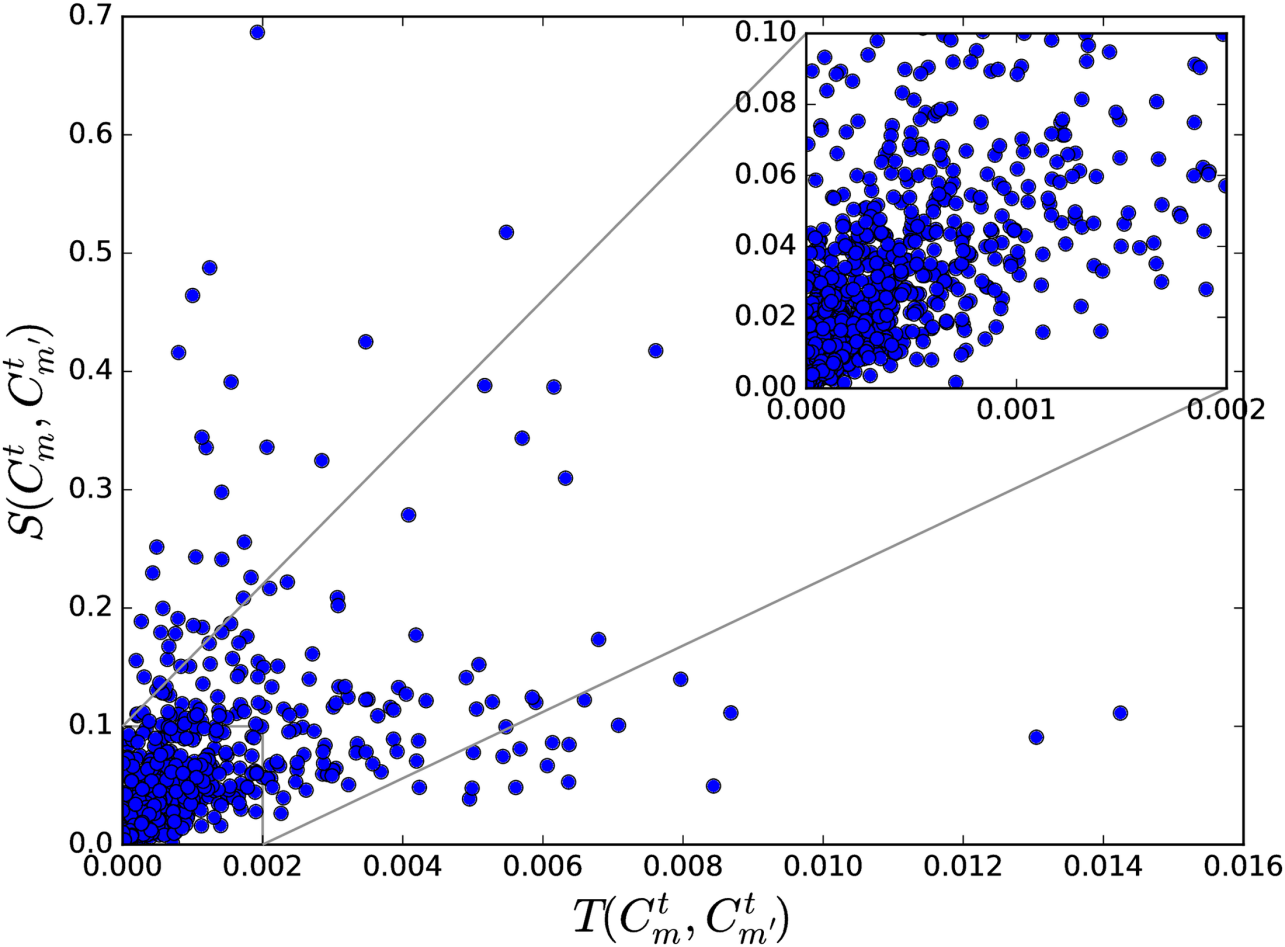
The scatter plot between $T(C_{m}^{t},C_{m^{\prime }}^{t})$ and $S(C_{m}^{t},C_{m^{\prime }}^{t})$ among all TCs (with at least 100 papers) in 1990s.

Next, we consider the Kuhnian processes of splitting and merging. For merging events, the similarity

$$\begin{array}{c} S\left(C_{m}^{t},C_{m^{\prime }}^{t}\right)=\sum_{n}\left(I_{mn}^{f}/\sum_{n^{\prime }}I_{mn^{\prime }}^{f}\right)\left(I_{m^{\prime }n}^{f}/\sum_{n^{\prime \prime }}I_{m^{\prime }n^{\prime \prime }}^{f}\right) \end{array}$$ (4)

measures the overlap between the offsprings of the TCs $C_{m}^{t}$ and $C_{m^{\prime }}^{t}$ in year *t*. If $C_{m}^{t}$ and $C_{m^{\prime }}^{t}$ merge perfectly into a single TC in year *t* + 1, *S* = 1. On the other hand, if the offsprings of $C_{m}^{t}$ and $C_{m^{\prime }}^{t}$ are distinct, *S* = 0. In general, 0 ≤ *S* ≤ 1. The value of *S* cannot be treated as a “prediction”, because we made use of information from years *t* and *t* + 1 to compute it. As two TCs evolved from being distinct to merging into a single TC, we expect to find few, low-weight edges between them in the BCN when they are distinct. This sum of weight of edges would gradually increase until the sum of weight between $C_{m}^{t}$ and $C_{m^{\prime }}^{t}$ is comparable to the sum of edges within $C_{m}^{t}$ and $C_{m^{\prime }}^{t}$. At this point, the two TCs merge. Therefore, to do the prediction, we define

$$\begin{array}{c} T\left(C_{m}^{t},C_{m^{\prime }}^{t}\right)=W\left(C_{m}^{t},C_{m^{\prime }}^{t}\right)/\left(L\left(C_{m}^{t}\right)L\left(C_{m^{\prime }}^{t}\right)\right), \end{array}$$ (5)

where $W(C_{m}^{t},C_{m^{\prime }}^{t})$ is the sum of weights of edges between papers in $C_{m}^{t}$ and $C_{m^{\prime }}^{t}$, normalized against the sizes of TCs involved. Fig 10 shows that $S(C_{m}^{t},C_{m^{\prime }}^{t})$ and $T(C_{m}^{t},C_{m^{\prime }}^{T})$ are highly correlated. High $T(C_{m}^{t},C_{m^{\prime }}^{t})$ leads with a large probability to a high $S(C_{m}^{t},C_{m^{\prime }}^{t})$. Analyzing the APS papers in the 1990s, we found a Spearman’s rank coefficient of 0.804 between $T(C_{m}^{t},C_{m^{\prime }}^{t})$ and $S(C_{m}^{t},C_{m^{\prime }}^{t})$ over all TCs (with at least 100 papers). However, because the average Pearson correlation coefficient is only 0.504, such a relation is not linear (see Fig 11).

**Fig 12:**
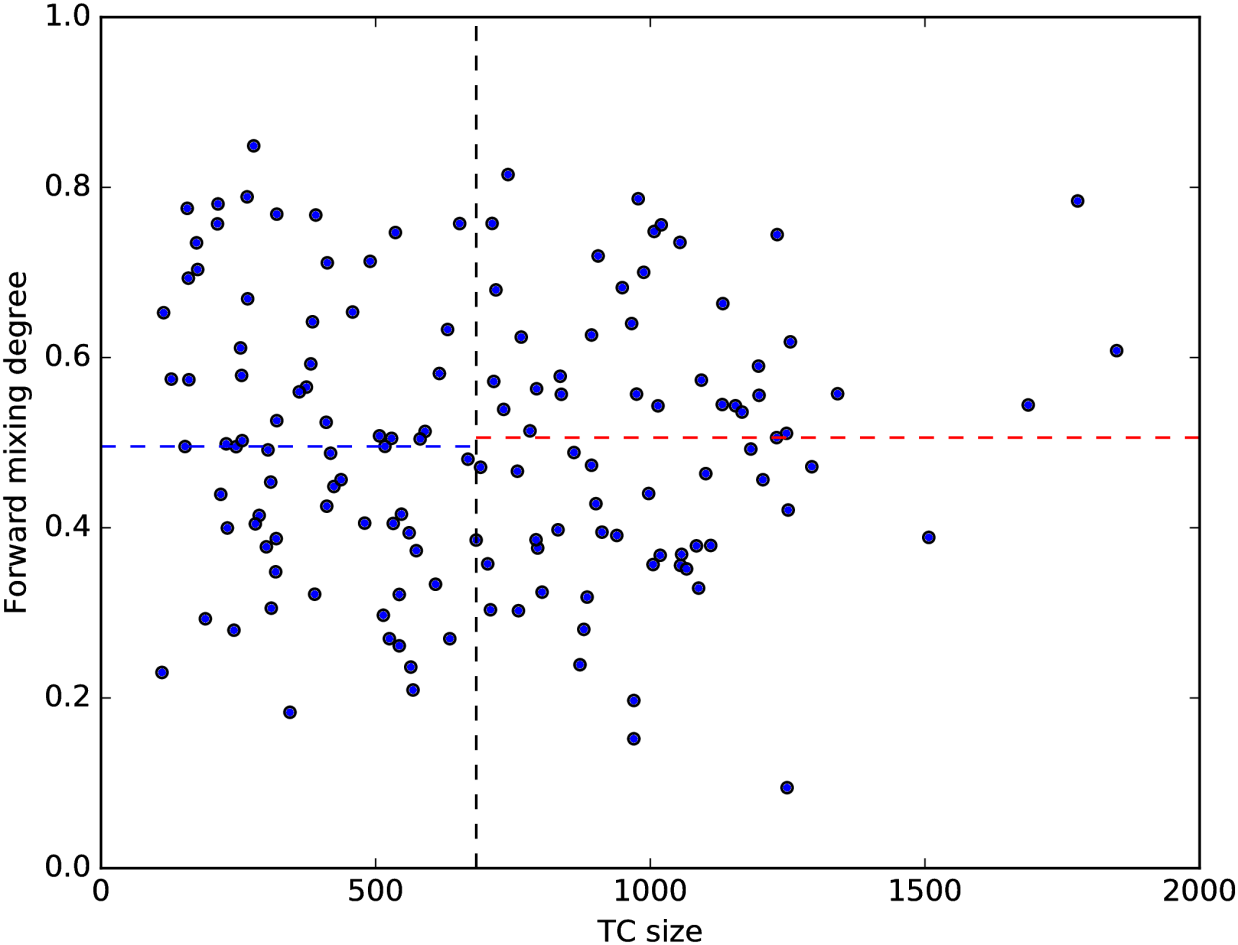
The scatter plot between size and forward mixing degree among all TCs (with at least 100 papers) in 1990s. The black dash line is median size, the blue dash line is the median of forward mixing degree for TCs are smaller than median size, the red dash line is the median of forward mixing degree for TCs are larger than median size.

**Fig 13:**
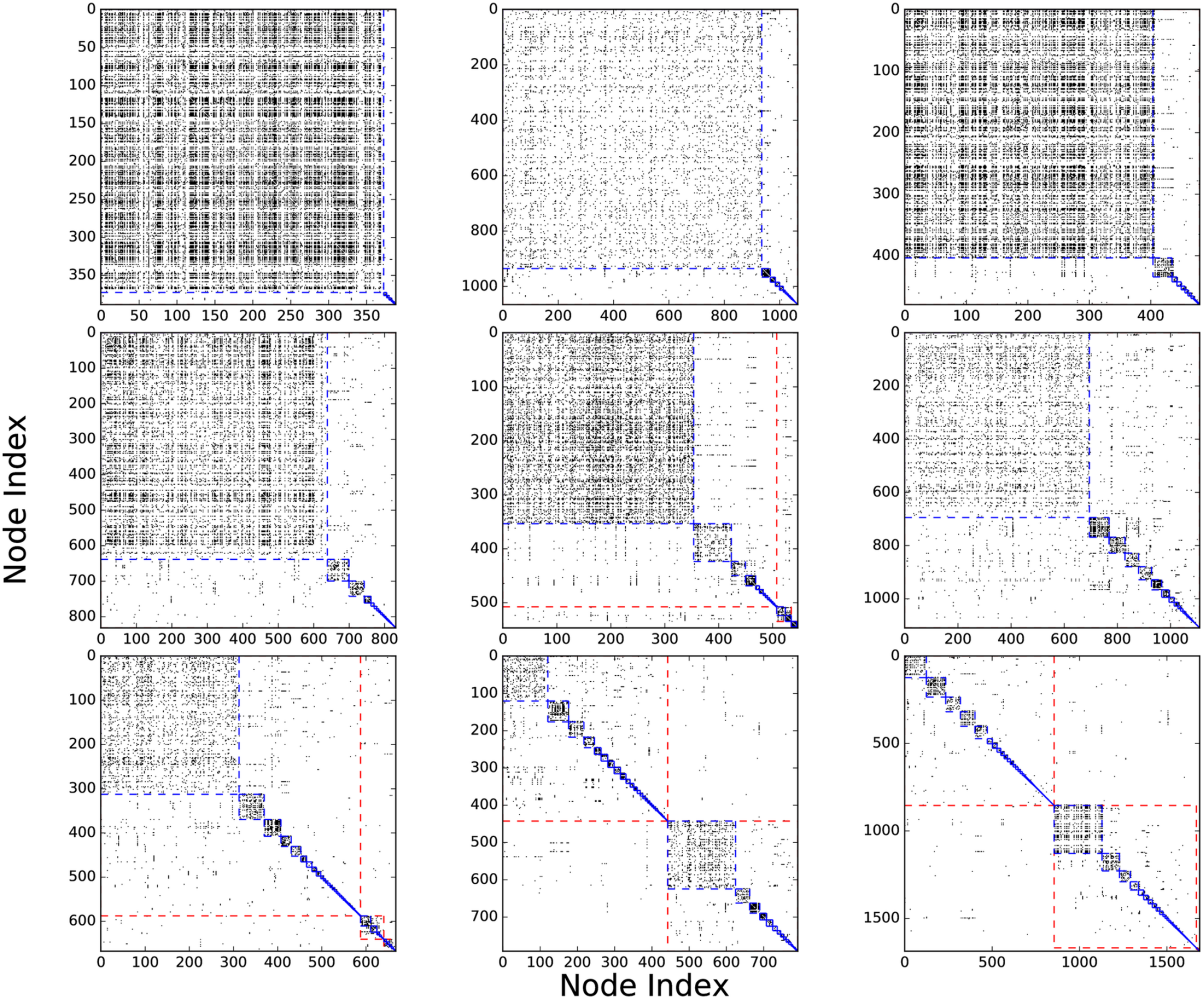
Adjacency matrices of TCs in the 1990s. The blue lines indicate the boundaries of subsubcommunities, the red lines indicate the boundaries of subcommunities. The red lines are absent from some plots because such TC have only one level when the Louvain algorithm terminated.

**Fig 14:**
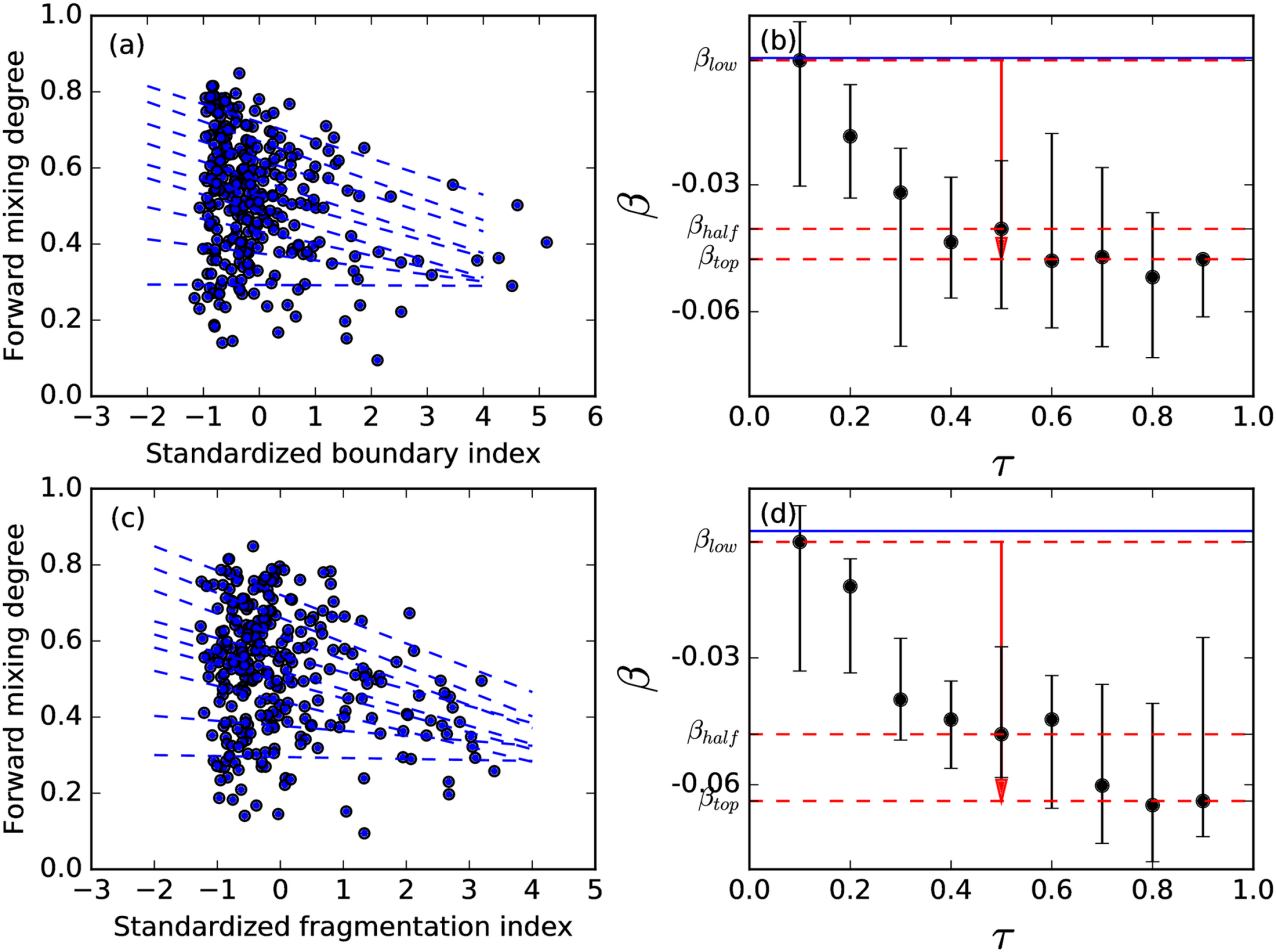
Relation between boundary index, fragmentation index and forward mixing degree of TCs in 1980s and 1990s. (a) Each dot corresponds to one TC, dash lines show QR results for quantiles *τ* = 0.1, 0.2, …, 0.9. (b) *β* coefficients (slopes of QR in the (a) as a function of *τ*. The red arrows show *β*_*low*_ ≡ *β*(*τ* = 0.1), *β*_*half*_ ≡ *β*(*τ* = 0.5) and *β*_*top*_ ≡ *β*(*τ* = 0.9), as, respectively, the nock, a circle on the shaft, and the head of the arrow, the blue solid line represents 0. (c) Each dot corresponds to one TC, dash lines show QR results for quantiles *τ* = 0.1, 0.2, …, 0.9. (d) *β* coefficients (slopes of QR in the (c) as a function of *τ*. The red arrows show *β*_*low*_ ≡ *β*(*τ* = 0.1), *β*_*half*_ ≡ *β*(*τ* = 0.5) and *β*_*top*_ ≡ *β*(*τ* = 0.9), as, respectively, the nock, a circle on the shaft, and the head of the arrow, the blue solid line represents 0.

We also tried to predict the splitting events. The first factor we considered is TC’s size. We divided all TCs in 1990s into two groups: one for TCs larger than median size, another for TCs smaller than median size. The medians and means for the forward mixing degree in these two groups are very close. Furthermore, the Pearson correlation coefficient between size and forward mixing degree is −0.031 (see Fig 12). Therefore, we concluded that a TC does not become more likely to split as it grows larger. The second factor is the TC’s internal structure. Here the situation is more complex: when we use the dendrogram extracted from the Louvain method to identify subcommunities, we found that different TCs have different internal structures (see Fig 13), some have a few large subcommunities, while others have many small subcommunities. Naively, we expect the criterion for splitting is the opposite to merging, i.e. the easier it is to tell one subcommunity from others, the higher the chances for a split. The *boundary index*

$$\begin{array}{c} B=\frac{\sum_{i_{1}\neq i_{2}}\sum_{\begin{array}{l} j_{1}\in C_{i_{1}}\\ j_{2}\in C_{i_{2}} \end{array}}A\left(j_{1},j_{2}\right)/\sum_{i_{i}\neq i_{2}}L\left(C_{i_{1}}\right)L\left(C_{i_{2}}\right)}{\sum_{i}\sum_{j_{1},j_{2}\in C_{i}}A\left(j_{1},j_{2}\right)/\sum_{i}L\left(C_{i}\right)L\left(C_{i}\right)}, \end{array}$$ (6)

which is the ratio between inter-subcommunity edge density and intra-subcommunity edge density, measures how indistinct the subcommunities are in a TC. Here *A*(*j*_1_, *j*_2_) is the weight of the edge between papers *j*_1_ and *j*_2_, and *C*_*i*_ is a subcommunity in the given TC. However the picture we find is not as simple as the merging case. When we plot *M*^*f*^ against *B*, we find the expected decreasing trend, but at the same time, the large scatter makes it impossible to reliably predict a splitting event using *B*. To better understand the relationship between *M*^*f*^ and *B*, we use quantile regression [21] to find that the *B* has no “prediction power” when *M*^*f*^ is small, but becomes “predictive” when *M*^*f*^ is large. That is to say the relation between *B* and *M*^*f*^ depends on the decile, as shown in Fig 14(a) and 14(b). The slopes show that for the decile of most strongly splitting TCs, increasing the standardized *B* by one standard deviation will decrease *M*^*f*^ by about 0.05, whereas for the decile of the least strongly splitting TCs, there is no obvious trend.

We also define a *fragmentation index*

$$\begin{array}{c} F=\sum_{i:j[i]}w_{i}S_{j[i]}^{2} \end{array}$$ (7)

where *w*_*i*_ is the size fraction of the top level subcommunity *i*, *s*_*j*[*i*]_ is the relative size fraction of subsubcommunity *j* inside subcommunity *i*. The more fragmented a community is, i.e., more and smaller subcommunities, the closer *F* is to 0. Quantile regression between *F* and *M*^*f*^ gives very similar results as *B* and *M*^*f*^, i.e., for the decile of most strongly splitting TCs, increasing the standardized *F* by one standard deviation will decrease *M*^*f*^ by about 0.06, whereas for the decile of the least strongly splitting TCs, there is no obvious trend as *β* close to 0, as shown in Fig 14(c) and 14(d).

**Fig 15:**
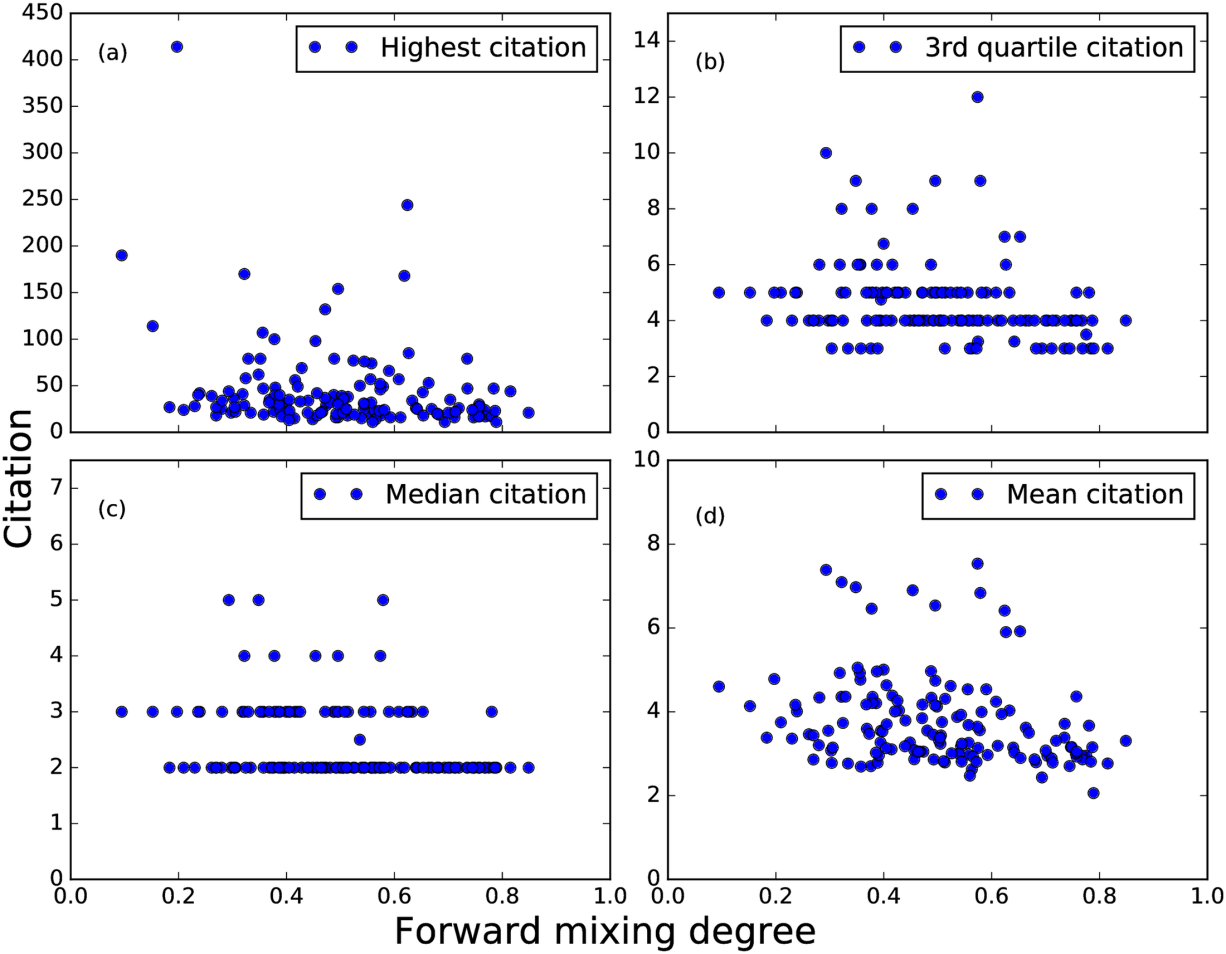
The scatter plot between different citations received during 2 years and forward mixing degree among all TCs (with at least 100 papers) in 1990s. (a) Highest citation, (b) Third quartile citation, (c) Median citation, (d) Mean citation.

Finally, we want to know the impacts of such merging and splitting events. We first check for an increase in the number of publications after such events, but found an insignificant difference in paper numbers in strongly and weakly mixing TCs (see Fig 9(b) and 9(c)). We suspected this is because our data set is confined to the APS publications, and a more careful check should include other physics journals to capture any “influence spillover”. When we think of high-impact research, we also think of highly-cited papers. Therefore, to quantify the impact of strongly-splitting events in the alluvial diagrams, we counted the citations of TCs resulting from splittings. As shown in Fig 15, we did this for number of citations 2 years after the events, and also 5 years after the events. There were no obvious trends. The results of backward mixing degree, i.e. merging, are similar.

Focusing on the highly productive chain of knowledge processes that led to experimental realizations of BEC, quantum teleportation and slow light, we checked the citation profiles between 1995 and 1998. While the 1995 BEC+QI+QO TC cited a slightly lower proportion of 1995 papers than the APS 0-year average, the 1996 BEC+QI+QO, the 1997 BEC TC, the 1998 BEC TCs all cited significantly more 0-year papers. The full effect of this BEC breakthrough can be seen in the large proportions of 1996 papers cited by the 1997 and 1998 TCs and the proportion of 1997 papers cited by the 1998 TC (see Fig 8). Indeed, we have provided early evidence suggesting that strongly-mixing Kuhnian processes are associated with greater impact.

## Conclusions

In this paper we have studied the knowledge evolution in physics restricted to the APS citation network. We built yearly bibliographic coupling networks, identified TCs and calculated the intimacy indices between TCs in successive years. Based on these results, we visualized the long-term knowledge evolution in physics research in the form of alluvial diagrams. From these alluvial diagrams, we see not only the split of PR into PRA, PRB, PRC and PRD in 1970, and the split of PRA into PRA and PRE in 1993, but also complex interactions between QO, QI and BEC. Overall, we find that the sizes of TCs are governed by a simple linear combination law. We find that merging events are highly correlated with inter-TC connections, making it possible to statistically predict merging events, while splitting events are more complex, as they are weakly correlated with the sizes or internal structures of the TCs. Finally, we showed using the QO, QI, and BEC case study that a strong merging/splitting event will significantly alter the field’s reference structure. In contrast with the well-known Popperian/Kuhnian qualitative and descriptive picture of science, which relate natural phenomena and their explanations with the practices of scientists, our approach leverage on the large and relatively complete data set on scientific publications, to look at changes at a whole-of-science (whole-of-physics in this paper) level, to quantify the types of changes (birth, death, growth, decay, merge, split) and their impacts on scientific research.

We should emphasize that our results should still be interpreted cautiously because of a number of limitations. One obvious limitation is that our citation network is restricted to APS journals, which are prestigious journals and likely reflect the frontiers of physics research, but are incomplete in two ways. First, many important physics papers are published outside of the APS journals, like Nature, Science and Applied Physics Letters. These papers are clearly important components of physics research. The second limitation is that even for the APS papers, we only have citations pointing back to APS papers. This means that the BCN between APS papers is not complete. For example, if two APS papers cite the same Nature paper, but no APS paper in common, this information will not be captured in the data set. The distortion due to the network being incomplete is hard to estimate, because we are dealing not only with missing nodes, but also with missing edges. There is also a third limitation, not due to the choice of the APS data set. This has to do with our choice to only consider pair-wise relations on merging prediction for simplicity. This is appropriate as a first step, but may not accurately reflect the many multiple-party merging events that we also see in our study.

While we believe we have made the correct first step towards a quantitative science of knowledge evolution, many open questions remain. The most crucial one is on the microscopic mechanisms governing merging and splitting. Can we build a model to simulate such evolution? How will this evolution picture change if we use more complete data set, like the Web of Science? It is well known that citation conventions in different disciplines are very different. What is therefore the evolution picture in other disciplines, like biology or social science? We seek to address these questions and more in future investigations.

## Supporting information

S1 File Case study: Quantum optics, quantum information and Bose-Einstein condensation. (PDF)
