# Supplementary material for: Knowledge evolution in physics research: An analysis of bibliographic coupling networks
Source: PLoS One. 2017 Sep 18;12(9):e0184821. doi: 10.1371/journal.pone.0184821 (PMC5602641; doi:10.1371/journal.pone.0184821)
Supplement: S1 File — (PDF) [file pone.0184821.s001.pdf]

Knowledge Evolution in Physics Research: An Analysis of Bibliography Coupling Networks
Supporting Information
Case study: quantum optics, quantum information and Bose-Einstein condensation

Wenyuan Liu(刘文源), Andrea Nanetti, and Siew Ann Cheong

To illustrate the utility our knowledge evolution framework can offer, we use as a case study the interesting interactions between quantum optics (QO), quantum information (QI), and Bose-Einstein Condensation (BEC). These three fields experienced breakthroughs in the 1990s. Table I shows the three most cited papers in these TCs, which are highlighted in Fig. 6. Key merging and splitting events are reported in the main paper, as are important publications these events are correlated with.

TABLE I: The three most cited papers in quantum optics, quantum information theory, quantum computation and Bose-Einstein condensation related TCs.

Year	TC	DOI	Title
1991	Upper	10.1103/PhysRevLett.67.661	Quantum cryptography based on Bells theorem
		10.1103/PhysRevLett.66.2593	Observation of electromagnetically induced transparency
		10.1103/PhysRevLett.67.1855	Enhancement of the index of refraction via quantum coherence
	Lower	10.1103/PhysRevA.44.5674	Above-surface neutralization of highly charged ions: The classical over-the-barrier model
		10.1103/PhysRevB.43.13401	Strong magnetic x-ray dichroism in 2p absorption spectra of 3d transition-metal ions
		10.1103/PhysRevLett.66.2601	Dynamic stabilization of hydrogen in an intense, high-frequency, pulsed laser field
1992	Upper	10.1103/PhysRevLett.69.2881	Communication via one- and two-particle operators on Einstein-Podolsky-Rosen states
		10.1103/PhysRevLett.69.3314	Observation of the coupled exciton-photon mode splitting in a semiconductor quantum microcavity
		10.1103/PhysRevLett.68.580	Wave-function approach to dissipative processes in quantum optics
	Lower	10.1103/PhysRevLett.68.1943	X-ray circular dichroism as a probe of orbital magnetization
		10.1103/PhysRevLett.68.3535	High-order harmonic generation from atoms and ions in the high intensity regime
		10.1103/PhysRevLett.69.1383	Absorption of ultra-intense laser pulses
1993	Upper	10.1103/PhysRevLett.70.1895	Teleporting an unknown quantum state via dual classical and Einstein-Podolsky-Rosen channels
		10.1103/PhysRevA.47.4114	Threshold and resonance phenomena in ultracold ground-state collisions
		10.1103/PhysRevLett.70.1244	Measurement of the Wigner distribution and the density matrix of a light mode using optical homodyne tomography: Application to squeezed states and the vacuum
	Lower	10.1103/PhysRevLett.71.1994	Plasma perspective on strong field multiphoton ionization
		10.1103/PhysRevLett.70.1599	Above threshold ionization beyond the high harmonic cutoff
		10.1103/PhysRevLett.70.774	High-order harmonic generation in rare gases with a 1-ps 1053-nm laser
1994	Upper	10.1103/PhysRevA.50.67	Squeezed atomic states and projection noise in spectroscopy
		10.1103/PhysRevLett.72.3439	Statistical distance and the geometry of quantum states
		10.1103/PhysRevLett.73.58	Experimental realization of any discrete unitary operator
	Lower	10.1103/PhysRevA.49.2117	Theory of high-harmonic generation by low-frequency laser fields
		10.1103/PhysRevLett.73.1227	Precision Measurement of Strong Field Double Ionization of Helium
		10.1103/PhysRevA.50.1540	Modeling harmonic generation by a zero-range potential
1995		10.1103/PhysRevLett.75.3969	Bose-Einstein Condensation in a Gas of Sodium Atoms
		10.1103/PhysRevLett.74.4091	Quantum Computations with Cold Trapped Ions
		10.1103/PhysRevA.52.R2493	Scheme for reducing decoherence in quantum computer memory
1996		10.1103/PhysRevA.54.3824	Mixed-state entanglement and quantum error correction
		10.1103/PhysRevLett.77.1413	Separability Criterion for Density Matrices
		10.1103/PhysRevLett.77.2360	Collective Excitations of a Trapped Bose-Condensed Gas
1997	Upper	10.1103/PhysRevLett.78.985	Bose-Einstein Condensation of Lithium: Observation of Limited Condensate Number
		10.1103/PhysRevLett.78.586	Production of Two Overlapping Bose-Einstein Condensates by Sympathetic Cooling
		10.1103/PhysRevLett.78.5	Demonstration of the Casimir Force in the 0.6 to $6mum$ Range
	Lower	10.1103/PhysRevLett.78.5022	Entanglement of a Pair of Quantum Bits
		10.1103/PhysRevLett.78.3221	Quantum State Transfer and Entanglement Distribution among Distant Nodes in a Quantum Network
		10.1103/PhysRevLett.79.3306	Noiseless Quantum Codes
1998	Upper	10.1103/PhysRevLett.81.3108	Cold Bosonic Atoms in Optical Lattices
		10.1103/PhysRevLett.81.938	Atomic Scattering in the Presence of an External Confinement and a Gas of Impenetrable Bosons
		10.1103/PhysRevLett.81.742	Spinor Bose Condensates in Optical Traps
	Lower	10.1103/PhysRevA.57.120	Quantum computation with quantum dots
		10.1103/PhysRevLett.80.2245	Entanglement of Formation of an Arbitrary State of Two Qubits
		10.1103/PhysRevLett.81.5932	Quantum Repeaters: The Role of Imperfect Local Operations in Quantum Communication
1999	Upper	10.1103/PhysRevLett.83.2498	Vortices in a Bose-Einstein Condensate
		10.1103/PhysRevLett.83.5198	Dark Solitons in Bose-Einstein Condensates
		10.1103/PhysRevLett.82.1975	Entanglement of Atoms via Cold Controlled Collisions
	Middle	10.1103/PhysRevLett.83.4204	Quantum Information Processing Using Quantum Dot Spins and Cavity QED
		10.1103/PhysRevB.59.2070	Coupled quantum dots as quantum gates
		10.1103/PhysRevLett.82.2417	Dynamical Decoupling of Open Quantum Systems
	Lower	10.1103/PhysRevLett.82.5229	Ultraslow Group Velocity and Enhanced Nonlinear Optical Effects in a Coherently Driven Hot Atomic Gas
		10.1103/PhysRevLett.83.2845	Transmission Resonances on Metallic Gratings with Very Narrow Slits
		10.1103/PhysRevLett.83.967	Liquid-Crystal Photonic-Band-Gap Materials: The Tunable Electromagnetic Vacuum
2000	Upper	10.1103/PhysRevLett.84.806	Vortex Formation in a Stirred Bose-Einstein Condensate
		10.1103/PhysRevLett.85.1795	Stable 85Rb Bose-Einstein Condensates with Widely Tunable Interactions
		10.1103/PhysRevLett.85.3745	Regimes of Quantum Degeneracy in Trapped 1D Gases
	Middle	10.1103/PhysRevA.62.062314	Three qubits can be entangled in two inequivalent ways
		10.1103/PhysRevLett.84.2722	Inseparability Criterion for Continuous Variable Systems
		10.1103/PhysRevA.62.012306	Electron-spin-resonance transistors for quantum computing in silicon-germanium heterostructures
	Lower	10.1103/PhysRevLett.85.5214	Double Resonant Raman Scattering in Graphite
		10.1103/PhysRevLett.85.154	Electronic Structure of Deformed Carbon Nanotubes
		10.1103/PhysRevB.62.13104	Carbon nanotubes, buckyballs, ropes, and a universal graphitic potential
